# Supplementary figures and images for: Experimental and computational investigation of enzyme functional annotations uncovers misannotation in the EC 1.1.3.15 enzyme class
Source: PLoS Comput Biol. 2021 Sep 23;17(9):e1009446. doi: 10.1371/journal.pcbi.1009446 (PMC8491902; doi:10.1371/journal.pcbi.1009446)

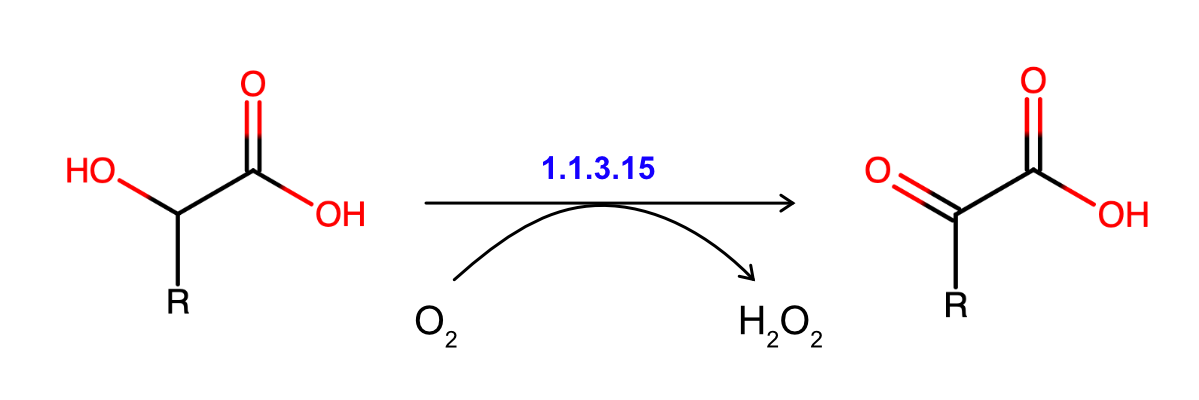

Supplement: S1 Fig — (TIFF) [file pcbi.1009446.s001.tiff]

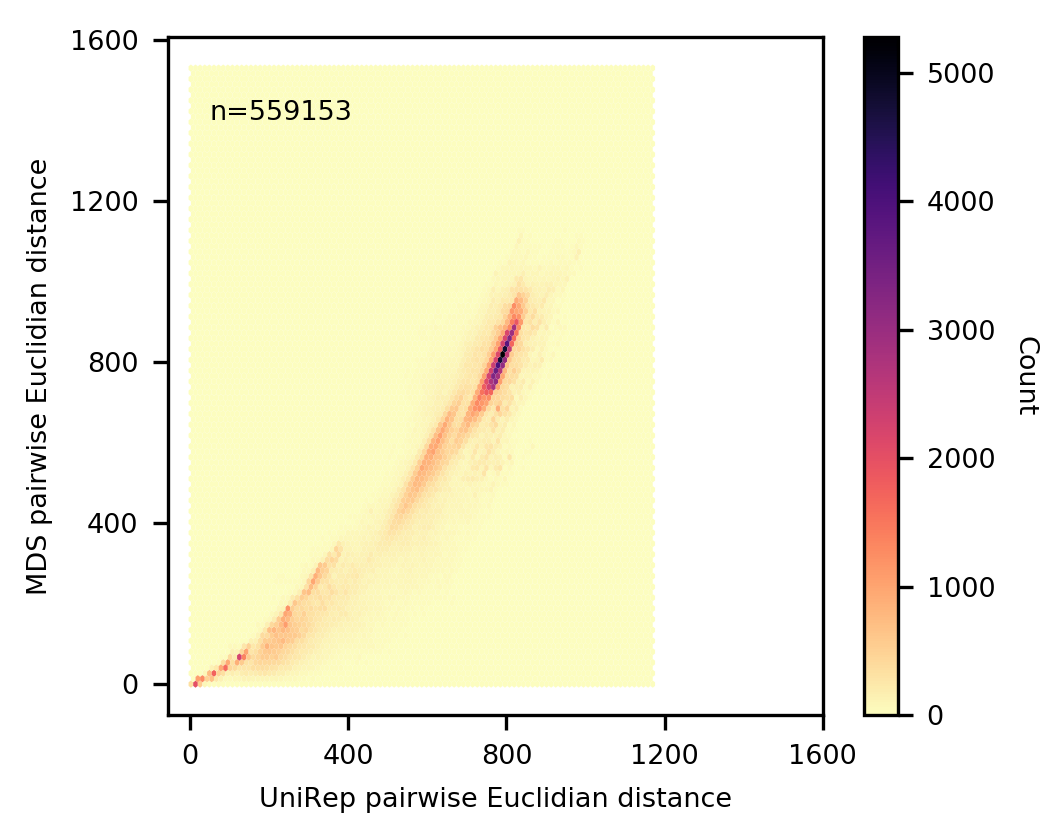

Supplement: S2 Fig — Clustering along the diagonal indicates that the multidimensional scaling (MDS) dimensionality reduction faithfully represents pairwise distances of the UniRep representations of these sequences. The total number of pairwise distances is indicated, corresponding to half of the distance matrix, without the diagonal. (TIFF) [file pcbi.1009446.s002.tiff]

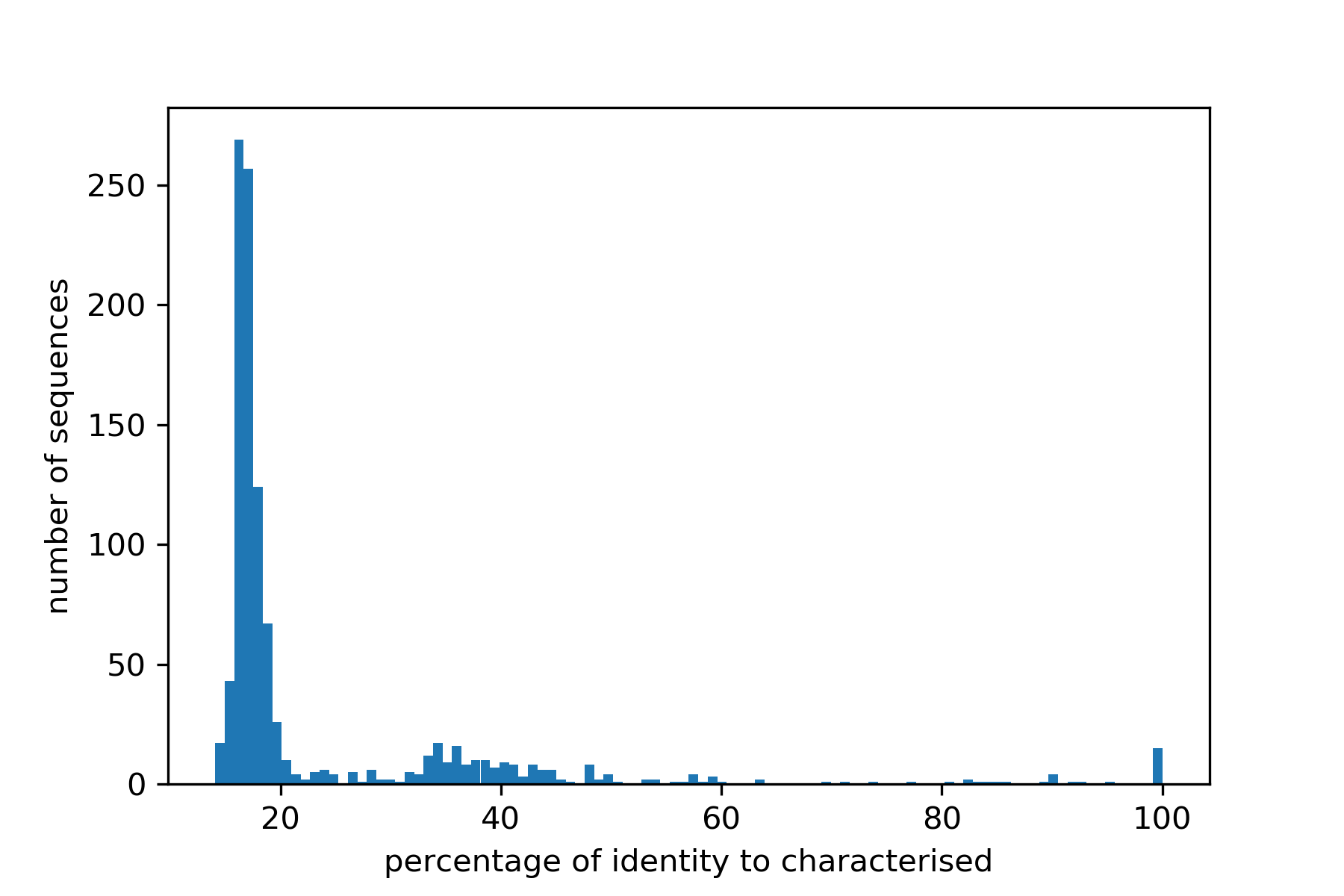

Supplement: S3 Fig — (TIFF) [file pcbi.1009446.s003.tiff]

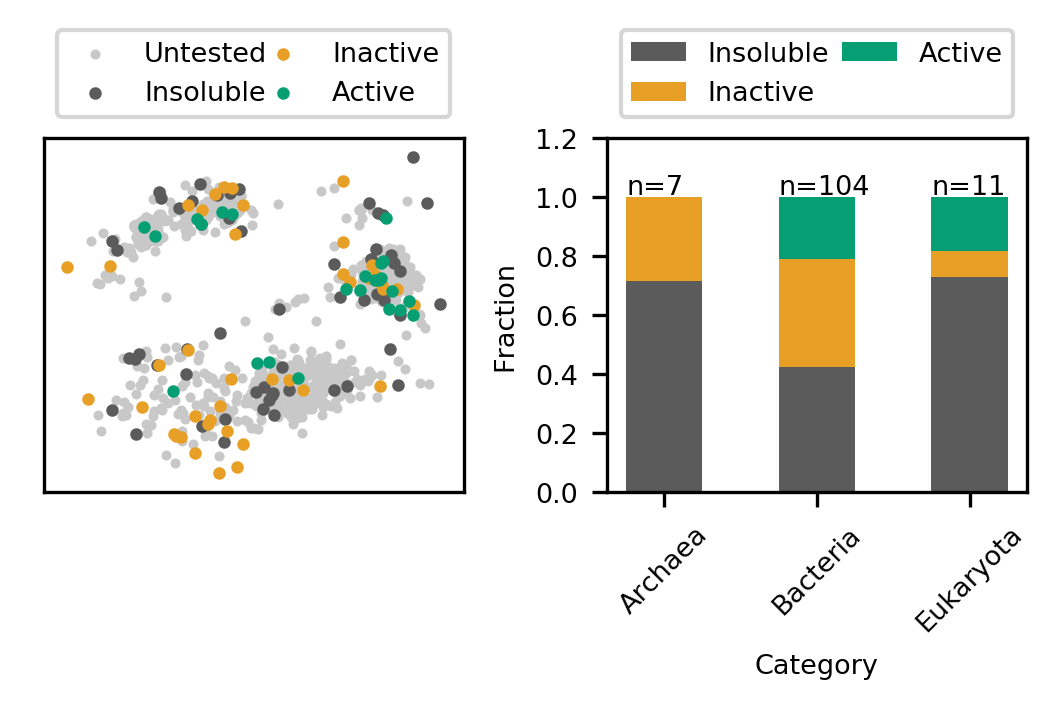

Supplement: S4 Fig — (TIFF) [file pcbi.1009446.s004.tiff]

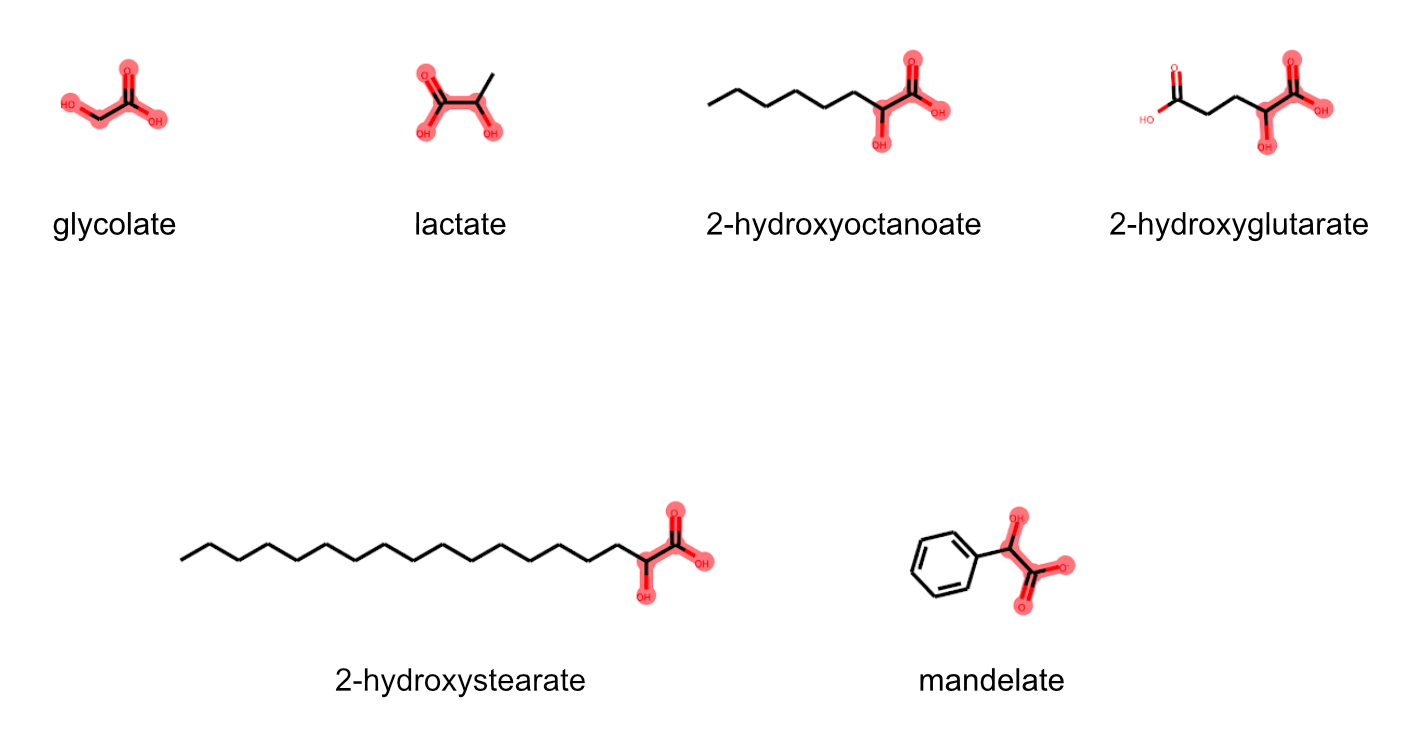

Supplement: S5 Fig — The donor group is marked in red. (TIFF) [file pcbi.1009446.s005.tiff]

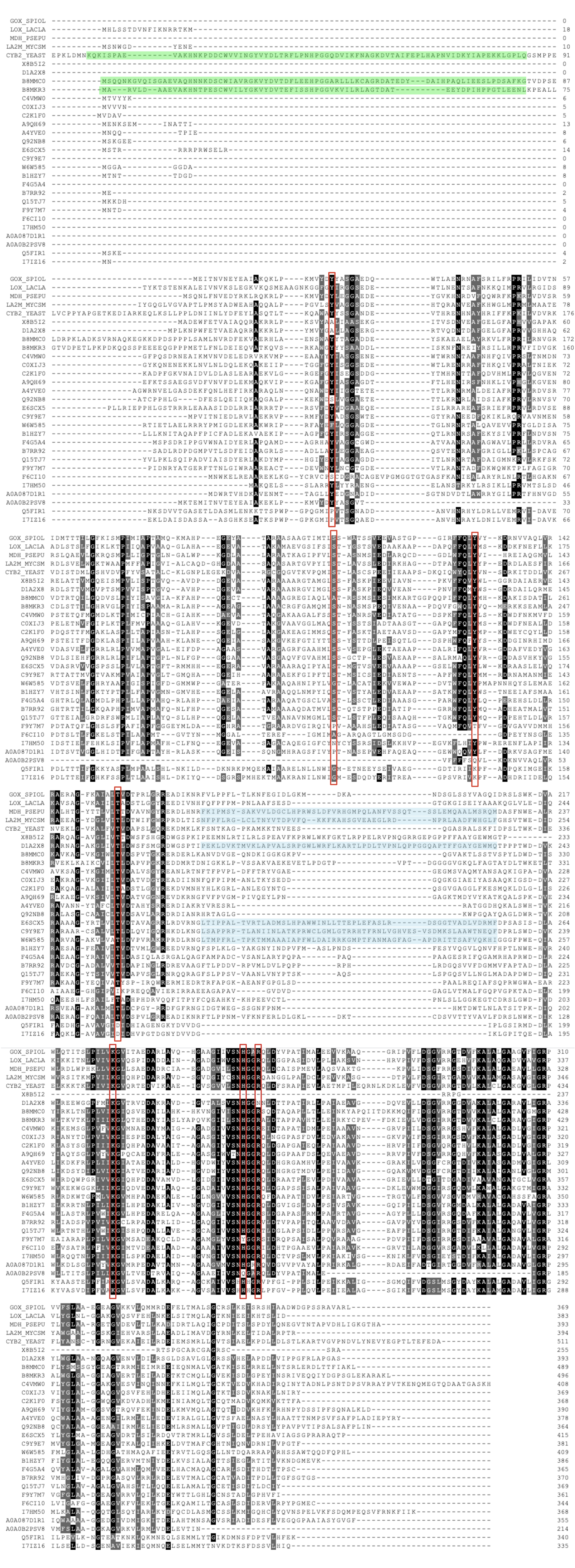

Supplement: S6 Fig — Conserved residues around the active site are circled in red. Sequence of predicted heme binding domain is highlighted in green, the elongated loop 4 is highlighted in blue. MSA performed in PROMALS3D (1) and visualised with Multiple Align Show (https://bioinformatics.org/sms/). (TIFF) [file pcbi.1009446.s006.tiff]

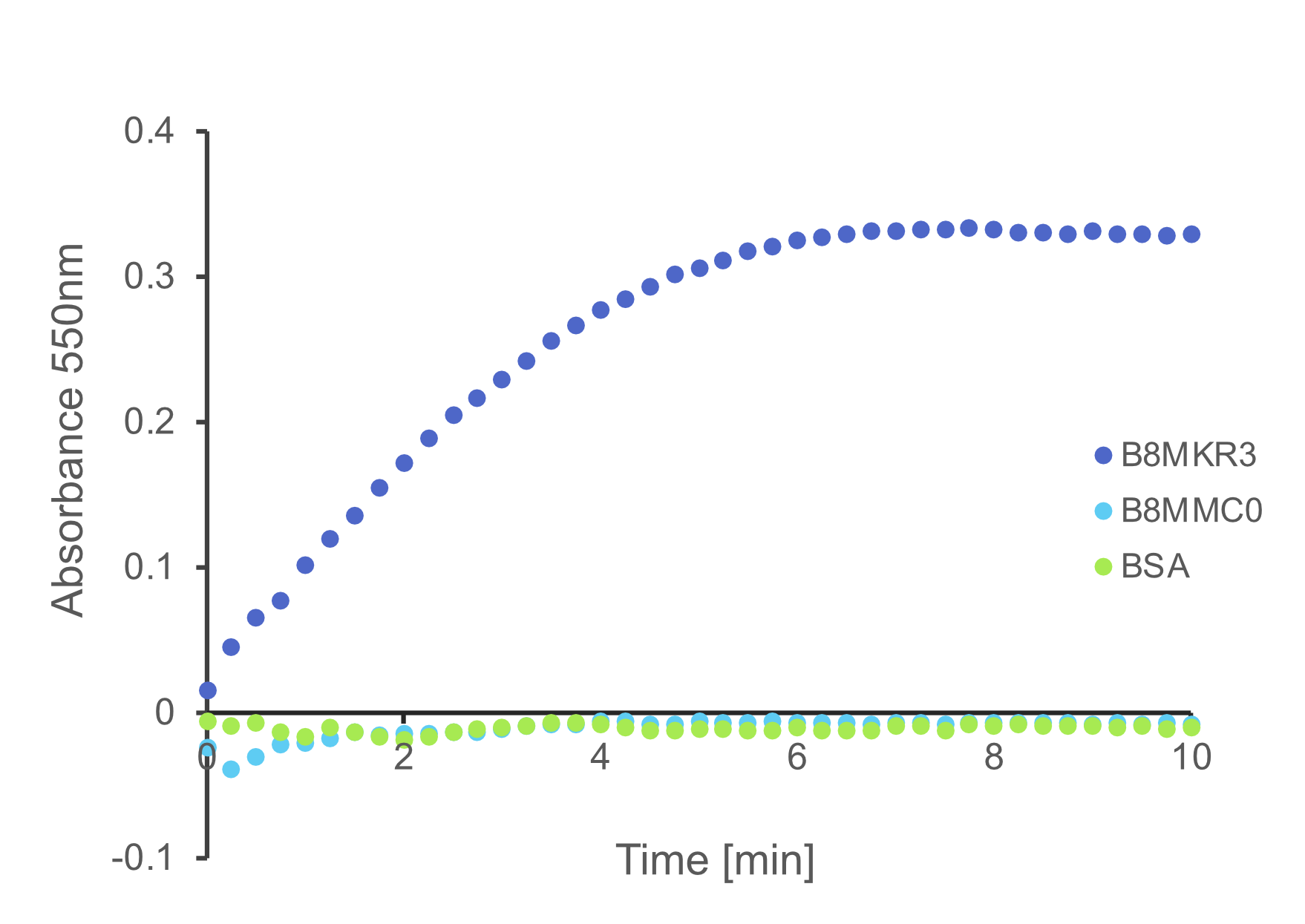

Supplement: S7 Fig — Increase of signal at the wavelength of 550 nm indicates reduction of cytochrome c and protein activity. (TIFF) [file pcbi.1009446.s007.tiff]

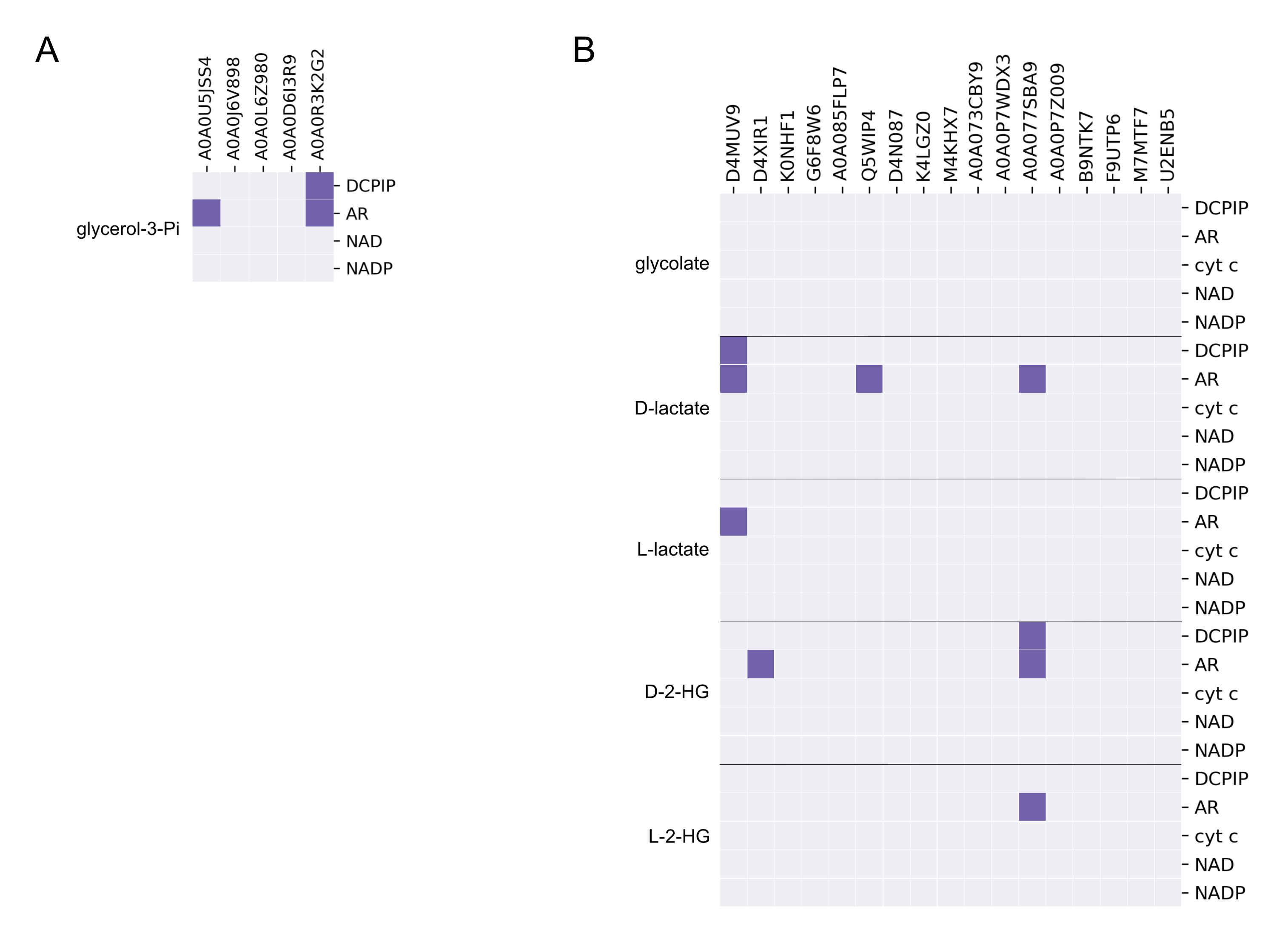

Supplement: S8 Fig — Presence of activity is marked with a dark purple square. (A) glycerol-3-phosphate dehydrogenase activity screen (B) 2-hydroxyglutarate dehydrogenase activity screen. (TIFF) [file pcbi.1009446.s008.tiff]

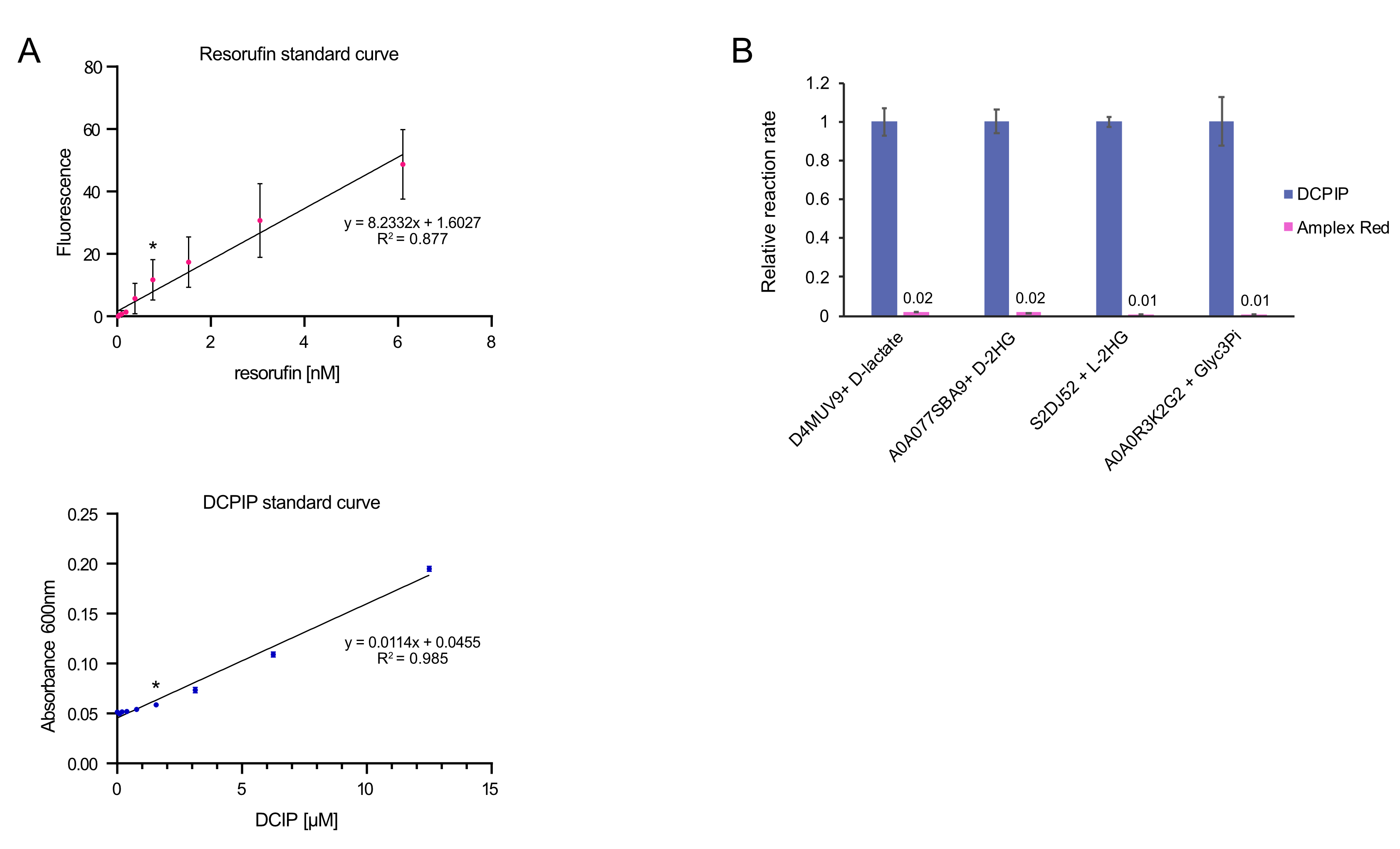

Supplement: S9 Fig — (A) Standard curves of resorufin, a product of Amplex Red-based assay (upper panel) and DCPIP (lower panel). Indicated by asterisk are concentrations of detection limit, as calculated by Anova single factor test (0.76 nM resorufin, 1.56 μM DCPIP). (B) Reaction rates of selected enzymes with the two electron acceptors, normalised to the reaction rate with DCPIP. Error bars in all figures represent standard deviation of the data obtained with three replicates. (TIFF) [file pcbi.1009446.s009.tiff]

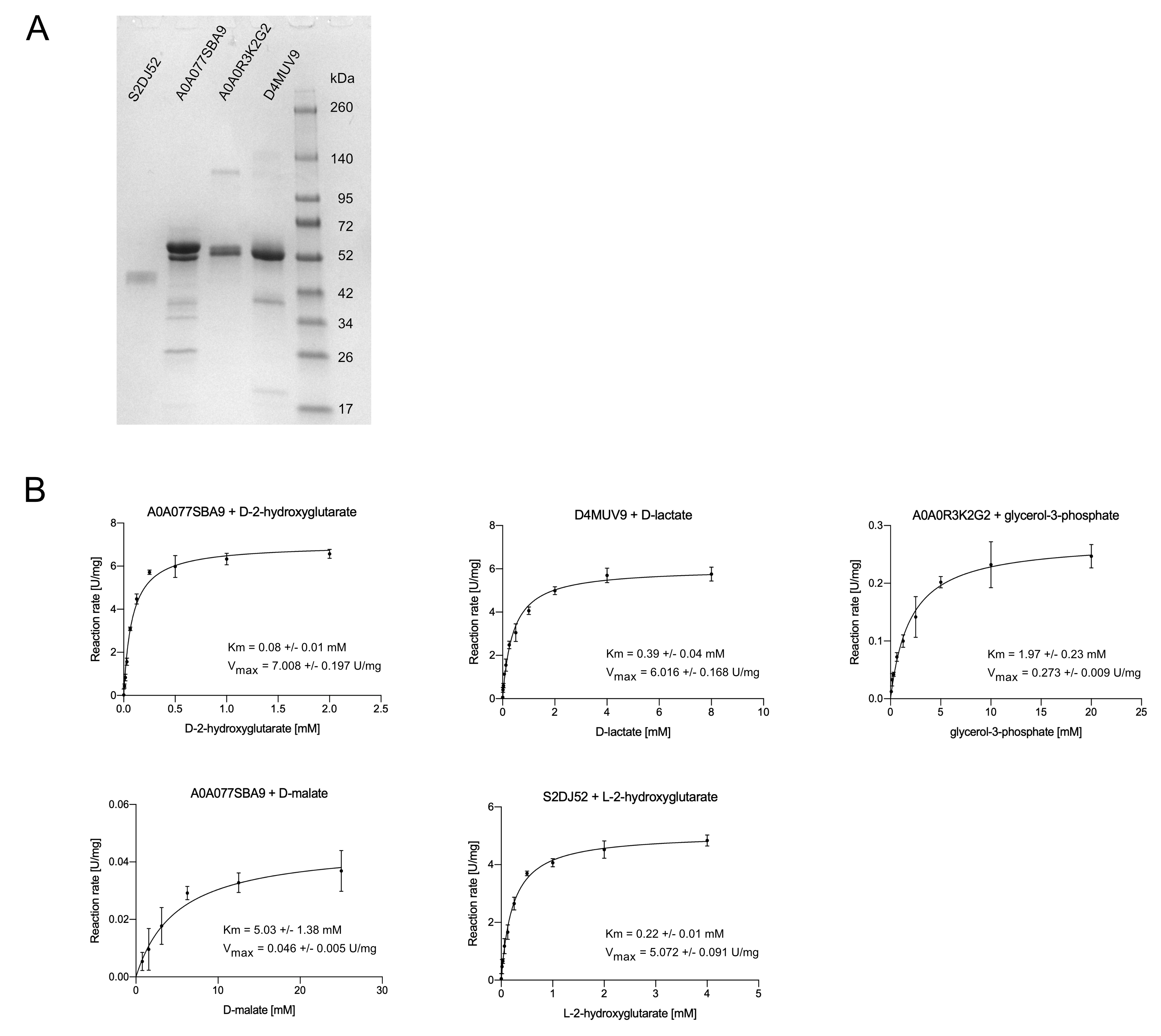

Supplement: S10 Fig — (A) SDS-PAGE gel of purified proteins chosen for kinetic characterisation. (B) Kinetic curves of the characterised enzymes. Error bars show standard error of three replicates. (TIFF) [file pcbi.1009446.s010.tiff]

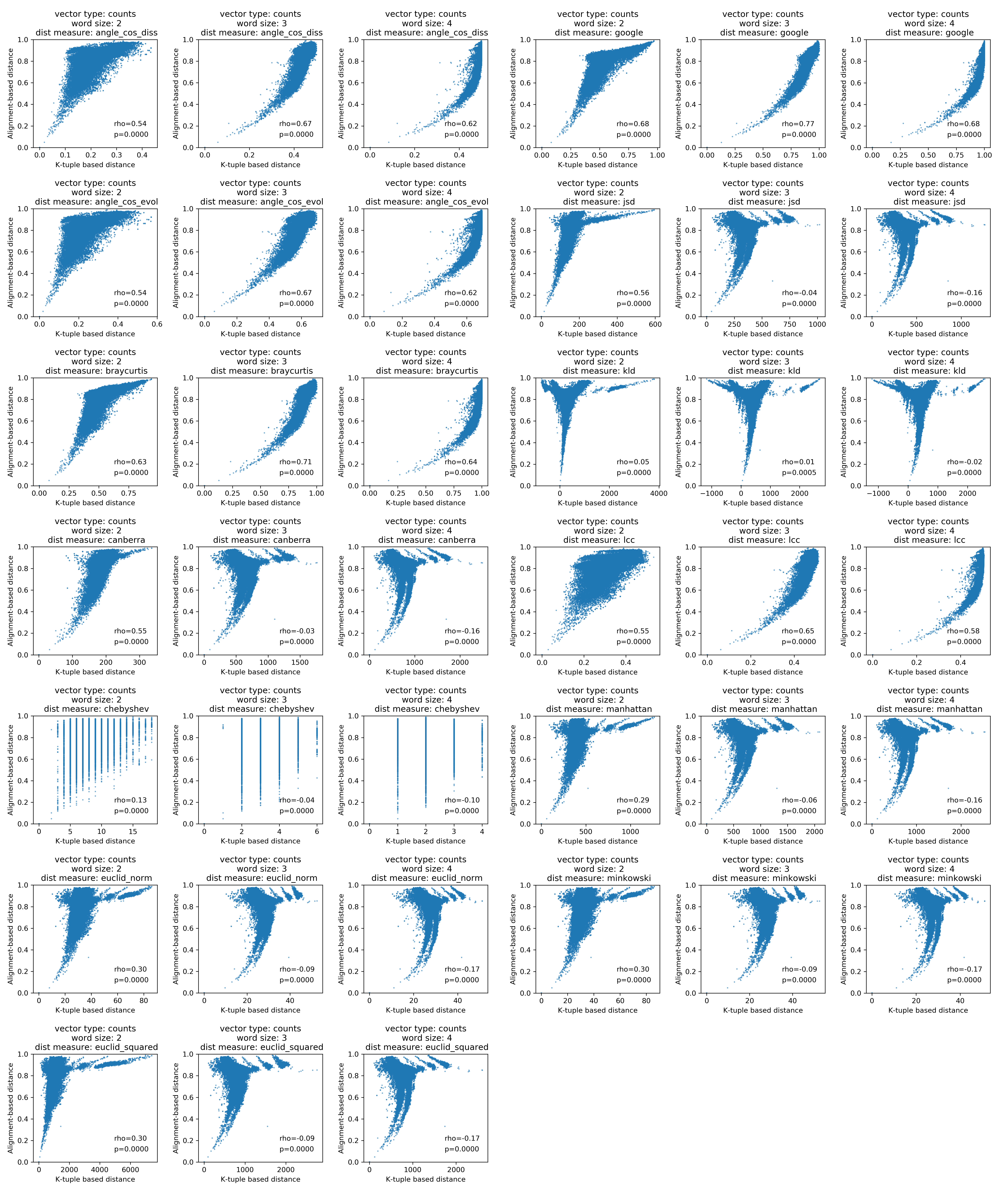

Supplement: S11 Fig — Using 400 randomly selected protein sequences all pairwise distances were calculated using different word size and distance measures. These distances were compared to distances computed using pairwise alignments. Appropriate k-tuple settings will cause points to lie on a diagonal, thus showing a high degree of correlation with the alignment-based values. Spearman’s rho and p-value is indicated for each plot. (TIFF) [file pcbi.1009446.s011.tiff]

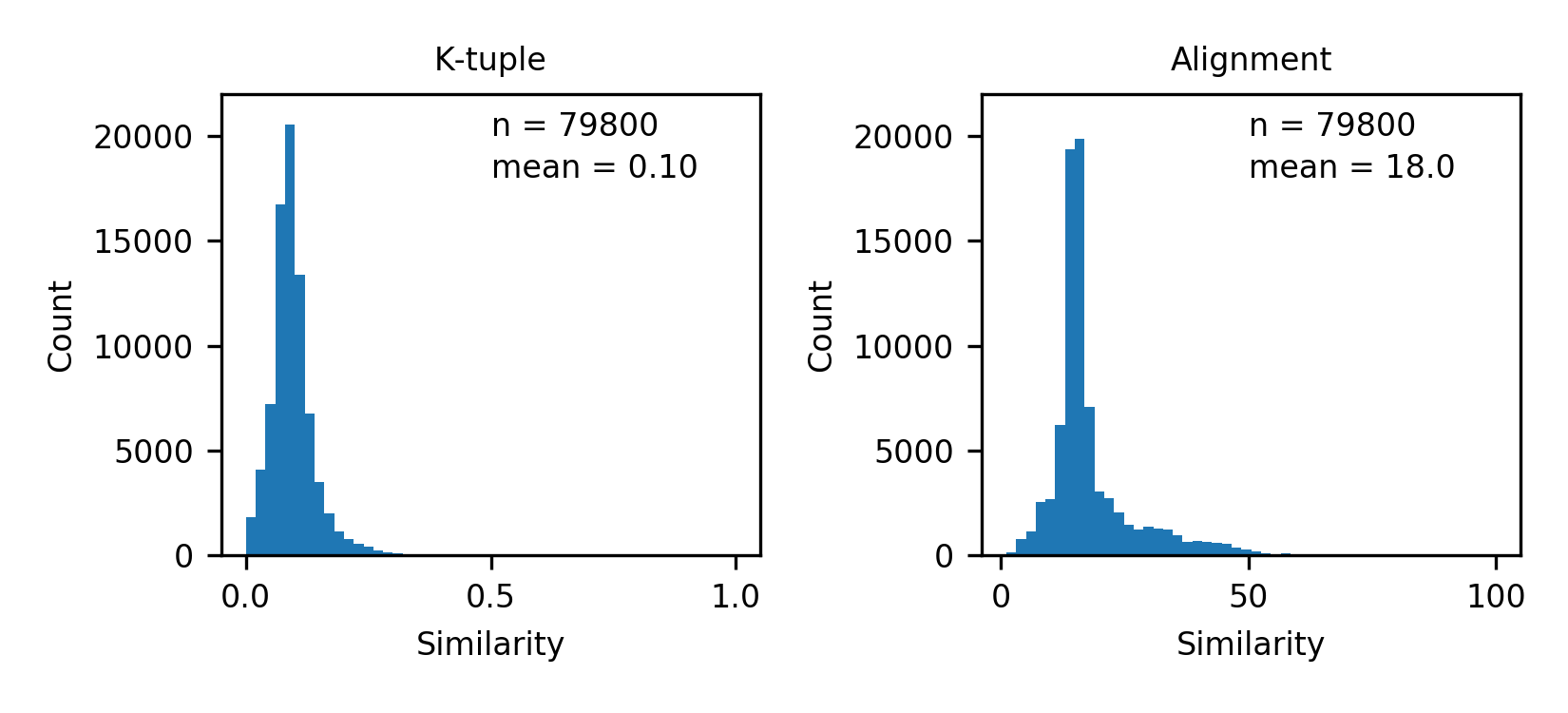

Supplement: S12 Fig — The k-tuple score was computed using a word size of 3 and google as a distance measure. The mean alignment-based identity is 18%. The total number of pairwise similarities is indicated, corresponding to half of the identity matrix, without the diagonal. (TIFF) [file pcbi.1009446.s012.tiff]
